# Acute Unilateral Peripheral Vestibulopathy After COVID-19 Vaccination: Initial Experience in a Tertiary Neurotology Center

**DOI:** 10.3389/fneur.2022.917845

**Published:** 2022-07-01

**Authors:** Marc Basil Schmid, David Bächinger, Athina Pangalu, Dominik Straumann, Julia Dlugaiczyk

**Affiliations:** ^1^Department of Otorhinolaryngology, Head and Neck Surgery, University Hospital Zurich, University of Zurich, Zurich, Switzerland; ^2^Department of Neuroradiology, University Hospital Zurich, University of Zurich, Zurich, Switzerland; ^3^Clinical Neuroscience Center, University Hospital Zurich, University of Zurich, Zurich, Switzerland; ^4^Department of Neurology, University Hospital Zurich, University of Zurich, Zurich, Switzerland

**Keywords:** acute unilateral peripheral vestibulopathy, vestibular neuritis, COVID-19, SARS-CoV-2, vaccination, herpes simplex virus, autoimmune cross-reactivity

## Abstract

**Objective:**

The aim of the present study was to identify patients who developed acute unilateral peripheral vestibulopathy (AUPVP) after COVID-19 vaccination.

**Methods:**

For this single-center, retrospective study, we screened the medical records of our tertiary interdisciplinary neurotology center for patients who had presented with AUPVP within 30 days after COVID-19 vaccination (study period: 1 June−31 December 2021). The initial diagnosis of AUPVP was based on a comprehensive bedside neurotological examination. Laboratory vestibular testing (video head impulse test, cervical and ocular vestibular evoked myogenic potentials, dynamic visual acuity, subjective visual vertical, video-oculography, caloric testing) was performed 1–5 months later.

**Results:**

Twenty-six patients were diagnosed with AUPVP within the study period. Of those, *n* = 8 (31%) had developed acute vestibular symptoms within 30 days after COVID-19 vaccination (mean interval: 11.9 days, SD: 4.8, range: 6–20) and were thus included in the study. The mean age of the patients (two females, six males) was 46 years (SD: 11.7). Seven patients had received the Moderna mRNA vaccine and one the Pfizer/BioNTech mRNA vaccine. All patients displayed a horizontal(-torsional) spontaneous nystagmus toward the unaffected ear and a pathological clinical head impulse test toward the affected ear on initial clinical examination. Receptor-specific laboratory vestibular testing performed 1–5 months later revealed recovery of vestibular function in two patients, and heterogeneous lesion patterns of vestibular endorgans in the remaining six patients.

**Discussion and Conclusions:**

The present study should raise clinicians' awareness for AUPVP after COVID-19 vaccination. The relatively high fraction of such cases among our AUPVP patients may be due to a certain selection bias at a tertiary neurotology center. Patients presenting with acute vestibular symptoms should be questioned about their vaccination status and the date of the last vaccination dose. Furthermore, cases of AUPVP occurring shortly after a COVID-19 vaccination should be reported to the health authorities to help determining a possible causal relationship.

## Introduction

Coronavirus disease 2019 (COVID-19) is a global pandemic caused by severe acute respiratory syndrome coronavirus 2 (SARS-CoV-2) with 489,678,203 confirmed cases and 6,149,250 deaths to date (2 April, 2022) (1). An unprecedented worldwide vaccination program has been rolled out since December 2020 to combat this devastating disease. The vaccines most commonly used in Switzerland, the European Union and the United States are based on mRNA technology (Cominarty® by Pfizer/BioNTech; Spikevax® by Moderna) or adenovirus vectors (COVID-19 vaccine Janssen® produced by Johnson and Johnson; Vaxzevria® by Oxford/AstraZeneca) (2) and induce an immune response against the spike glycoprotein of SARS-CoV-2, which is pivotal for viral invasion of host cells (3). Vaccination is an effective tool to prevent infection with the virus, to ameliorate the course of the disease and to reduce the death toll of COVID-19 (4, 5).

Since the beginning of the vaccination program, an increasing number of adverse medical events following administration of the vaccine has been reported. While temporal association does not prove causality, it is nevertheless important to make note of such cases and report them to the health authorities. Based on these data, large-scale post-authorization studies can be conducted to re-evaluate the safety of a vaccine and to re-weigh the risk-benefit-ratio of vaccinations for specific subgroups of the population (6, 7).

A wide spectrum of new-onset neurological disorders following COVID-19 vaccination has been reported so far, including disorders of the brain (e.g., venous sinus thrombosis, acute demyelinating encephalomyelitis), the spinal cord (acute transverse myelitis), the peripheral nervous system (Guillan-Barré syndrome), the muscles (myositis) and the cranial nerves (olfactory dysfunction, optic neuritis, abducens and facial nerve palsies) (8). Reports of sudden sensorineural hearing loss (SSNHL) and tinnitus following COVID-19 vaccination have raised the question whether the eighth cranial nerve and/or the inner ear might also be affected by processes following COVID-19 vaccination. Possible pathomechanisms discussed in this context comprise autoimmune cross-reactivity between pathogen and host proteins, deposition of immune complexes in the inner ear and reactivation of herpes simplex virus (HSV) or varizella zoster virus (VZV) in the ganglia of the eighth cranial nerve (9, 10).

While cochlear symptoms (e.g., SSNHL) have been described in several case reports and cohort studies [see (11) for summary], only two cases of acute unilateral peripheral vestibulopathy (AUPVP) following COVID-19 vaccination could be found in the medical literature so far (2 April, 2022) to the best of our knowledge (12, 13). AUPVP (often also called vestibular neuritis) is an acute vestibular syndrome (AVS) characterized by (i) a horizontal or horizontal-torsional spontaneous nystagmus (SN) following Alexander's law, beating toward the contralateral - i.e., unaffected - ear, (ii) a positive clinical head impulse test of the horizontal semicircular canal (h-HIT) toward the affected ear, (iii) and the absence of concomitant hearing loss or other neurological signs and symptoms (14). AUPVP affects endorgans of the superior vestibular nerve (lateral and anterior semicircular canals, utricle) more frequently than those of the inferior portion of the nerve (posterior semicircular canal, saccule) (15). It is not clear to date whether the disorder is caused by intralabyrinthine lesions, an inflammation of the nerve (“vestibular neuritis”) or both (16, 17). Possible underlying pathomechanisms include HSV reactivation, autoimmune response or microvascular ischemic insults to the vestibular labyrinth (18).

The case report about AUPVP after COVID-19 vaccination by Jeong (12) presents detailed results of the bedside neurotological examination and vestibular testing that allow to exclude other causes of AVS, while Canales Medina and Ramirez Gómez (13) only describe a spontaneous nystagmus without differentiating further between a peripheral and a central acute vestibular disorder, e.g., by application of the HINTS (head impulse, nystagmus, test of skew) paradigm (19). Likewise, many case reports about AUPVP or labyrinthitis in patients with SARS-CoV-2 infection are rather based on symptoms and exclusion of other disorders than on clinical and laboratory vestibular testing (20–23). Thus, it remains questionable whether the patients' vertigo was really caused by an acute unilateral peripheral vestibulopathy (24, 25).

Therefore, the aim of the present study was to identify patients with AUPVP after COVID-19 vaccination based on bedside neurotological examination and receptor-specific vestibular laboratory testing. These data are important to establish a realistic risk-benefit ratio of COVID-19 vaccines regarding vestibular disorders and, hence, are not intended to discourage vaccination (7, 9).

## Patients and Methods

This single-center, retrospective study was carried out at an interdisciplinary tertiary neurotology center. The main catchment area of our institution is the Kanton Zurich with currently 1.56 million inhabitants (26).

### Patients

Within the study period (1 Jun to 31 Dec, 2021), a total of 26 patients were diagnosed in our institution with AUPVP based on the following criteria: (i) horizontal or horizontal-torsional SN following Alexander's law, beating toward the contralateral - i.e., unaffected - ear, (ii) a positive h-HIT toward the affected ear, (iii) absence of concomitant hearing loss or other neurological signs and symptoms, and (iv) symptoms not better accounted for by any other vestibular disorder (14). Only those patients with AUPVP who had developed acute vestibular symptoms within 30 days after a COVID-19 vaccination (n = 8) were included into the present retrospective study (Table 1). Patients #2 and #6 had initially been diagnosed with AUPVP *alio loco* before they were referred to our center. The other six patients presented to us shortly after the acute onset of symptoms.

**Table 1:** Patient demographics and vaccination status.

**Patient nr**.	**Age (years)**	**Gender**	**Vaccine (date)**			**AUPVP onset**	**Interval vaccine - AUPVP**
			**1^**st**^dose**	**2^**nd**^dose**	**Booster**		
#1	37	m	Moderna (2 May, 2021)	Moderna (1 Jun, 2021)	–	7 Jun, 2021	6 days
#2	58	m	Moderna (12 May, 2021)	–	–	1 Jun, 2021	20 days
#3	35	m	Moderna (3 Jul, 2021)	**–**	**–**	12 Jul, 2021	9 days
#4	34	f	Moderna (26 Jun, 2021)	**–**	**–**	6 Jul, 2021	10 days
#5	44	m	Moderna (10 Jun, 2021)	Moderna (1 Jul, 2021)	–	7 Jul, 2021	7 days
#6	66	m	Moderna (7 May, 2021)	Moderna (4 Jun, 2021)	–	14 Jun, 2021	10 days
#7	53	m	Pfizer (14 Jan, 2021)	Pfizer (11 Feb, 2021)	Pfizer (27 Nov, 2021)	15 Dec, 2021	18 days
#8	53	f	Moderna (12 May, 2021)	Moderna (08 Jun, 2021)	Moderna (16 Dec, 2021)	31 Dec, 2021	15 days

“*age”, age at symptom onset; AUPVP, acute unilateral peripheral vestibulopathy; “interval”, time interval between last dose of COVID-19 vaccine and onset of AUPVP symptoms*.

The 30-day interval was based on an extensive literature research about the onset of the following disorders following a COVID-19 vaccination: (i) AUPVP and autoimmune vestibulopathy (2 days to 1 month) (12, 13, 27), (i.) sudden sensorineural hearing loss (0–30 days) (9, 11) (iii) HSV or VZV reactivation (1–16 days) (8, 28–31), and (iv) new-onset neurological disorders (0 days to 1 month) (8, 32).

Exclusion criteria for the present study were: (i) a positive history of SARS-CoV-2 infection before the onset of AUPVP, (ii) onset of acute vestibular symptoms before vaccination, (iii) acute onset of hearing loss after COVID-19 vaccination or (iv) better explanation of symptoms by another vestibular disorder. Of note, no patient presented to our institution with an acute cochleovestibular syndrome within 30 days after COVID-19 vaccination during the study period.

The study was carried out according to the recommendations of the Cantonal Ethics Committee Zurich and in accordance with the Declaration of Helsinki. All clinical and laboratory examinations were part of the clinical routine at our hospital. All patients included into the study provided prior informed consent.

### Bedside Neurotological Examination

On initial presentation with AVS, all patients except #2 and #6 (referral after diagnosis of AUPVP had been made *alio loco*) underwent a comprehensive bedside neurotological examination by a neurologist or an otorhinolaryngologist, as described by Straumann (33), including: examination of horizontal and vertical smooth pursuit and saccades; alternating cover test for detection of skew deviation; h-HIT; exploration of spontaneous, gaze-evoked and head-shaking nystagmus (with and without Frenzel goggles), bilateral Dix-Hallpike and supine roll maneuvers with Frenzel goggles; testing of cerebellar coordination (diadochokinesis, finger-to-nose test), Romberg and Romberg-on-foam test (“FRomberg”) for examination of postural control (Table 2). The HINTS paradigm (head impulse, nystagmus, test of skew) was applied in each patient to disclose a potential central origin of the AVS (19).

**Table 2:** Bedside neurotological examination on initial presentation and on follow-up examination (same day as laboratory vestibular testing, see Table 3).

**Patient nr**.	**SP**	**SAC**	**SD**	**h-HIT**	**SN**	**GEN**	**HSN**	**Positioning maneuvers**	**Cerebellar coordination**	**Romberg**	**FRomberg**	**Diagnosis**
#1 (initial)	Super-imposed by SN	N	no	**R**	**L II °** (Alexander's law, suppressed by fixation)	No	**L**	n.d.	N	**Sway to R**	n.d.	R AUPVP
#1 (1 month)	N	N	No	N	No (±Frenzel goggles)	No	No	N	N	N	N	Clinical recovery of R h-VOR
#2 (initial)	N	N	No	**R**	**L III**° (Alexander's law, suppressed by fixation)	No	**L**	**L nystagmus (like SN)** in all positions	N	**Not able to stand with eyes closed**	n.d.	R AUPVP
#2 (5 months)	N	N	No	**R**	no (±Frenzel goggles)	No	No	N	N	N	N	R h-VOR hypofunction
#3 (initial)	N	N	No	**R**	**L III**° (Alexander's law, suppressed by fixation)	No	**L**	n.d.	N	**sway to R**	n.d.	R AUPVP
#3 (1 month)	N	N	No	N	no (±Frenzel goggles)	No	No	N	N	N	N	Clinical recovery of R h-VOR
#4 (initial)	N	N	No	**R**	**L III**° (Alexander's law, suppressed by fixation)	No	**L**	**L nystagmus (like SN)** in all positions	N	**Sway to R**	n.d.	R AUPVP
#4 (1 month)	N	N	No	**R**	No (±Frenzel goggles)	No	**L**	N	N	N	N	R h-VOR hypofunction
#5 (initial)	N	N	No	**L**	**R** (Alexander's law, suppressed by fixation)	No	n.d.	n.d.	N	**Slight sway to L**	n.d.	L AUPVP
#5 (1 month)	N	N	No	**L**	no (±Frenzel goggles)	No	No	N	N	N	N	L h-VOR hypofunction
#6 (initial)	N	N	No	**L**	**R** (Alexander's law, suppressed by fixation)	No	**R**	n.d.	N	**sway to L**	n.d.	L AUPVP
#6 (4 months)	N	N	No	**L**	no (±Frenzel goggles)	No	**R**	N	N	N	N	L h-VOR hypofunction
#7 (initial)	N	N	No	**R**	**L III**° (Alexander's law, suppressed by fixation)	No	**L**	n.d.	N	**Not able to stand with eyes closed**	n.d.	R AUPVP
#7 (1 month)	N	N	No	**R**	**L II**° (only with Frenzel goggles)	No	**L**	N	N	N	n.d.	R h-VOR hypofunction
#7 (3 months)	N	N	No	N	no (±Frenzel goggles)	No	No	N	N	N	N	Clinical recovery of R h-VOR
#8 (initial)	N	N	No	**L**	**R III**° (Alexander's law, suppressed by fixation)	No	**R**	n.d.	N	**Sway to L**	n.d.	L AUPVP
#8 (1 month)	N	N	No	N	no (±Frenzel goggles)	No	No	n.d.	N	N	N	Clinical recovery of L h-VOR

*Pathological findings are printed in bold*.

*SP, smooth pursuit; SAC, saccades; SD, skew deviation; h-HIT, clinical head-impulse test for the horizontal semicircular canal (“R/L”: positive for head turns to the right/left side); SN, spontaneous nystagmus; GEN, gaze-evoked nystagmus; HSN, head-shake nystagmus; positioning maneuvers, Dix-Hallpike and supine roll maneuvers to both sides; N, normal; n.d., not done; AUPVP, acute unilateral peripheral vestibulopathy; h-VOR, vestibulo-ocular reflex of the horizontal semicircular canal*.

### Vestibular Laboratory Testing

After the diagnosis of AUPVP had been made, all patients were scheduled for clinical follow-up and vestibular laboratory testing after 1 month according to the routine clinical protocol of our institution for patients with AUPVP (Table 3). As patients #2 and #6 were referred from different healthcare providers, vestibular testing was performed with a latency of 5 (#2) and 4 months (#6) after the acute event. The following tests were applied for a detailed analysis of semicircular canal, utricular and saccular function.

**Table 3:** Vestibular laboratory testing 1 to 5 months after onset of acute peripheral vestibulopathy (AUPVP).

**Patient nr**.	**Side affected**	**vHIT gain**			**oVEMP AR**	**cVEMP AR**		**DVA**	**SVV**	**VOG (SPV)**	**CP**	**Interpretation (summary)**
		**HC**	**AC**	**PC**		**ACS**	**BCV**					
#1 (1 month)	R	1.0	0.9	0.7	−0.01	0.01	n.d.	0.1	−0.2°	**VIN R** (3°/sec) (recovery nystagmus)	n.d.	Recovery of R vestibular function
#2 (5 months)	R	**0.7** **(CS, OS)**	0.8	1.1	**0.42**	0.26	n.d.	**0.8**	**2.3**°	**VIN L** (3°/s)	19%	Hypofunction R HC and utricle
#3 (1 month)	R	1.1	1.0	1.3	−0.07	0.07	n.d.	0.2	−0.1°	no significant nystagmus	−14%	Recovery of R vestibular function
#4 (1 month)	R	**0.3** **(CS, OS)**	0.8	1.0	n.d.	n.d.	n.d.	**1.2**	**3.1**°	n.d.	n.d.	Hypofunction R HC and utricle (saccule not tested)
#5 (1 month)	L	**0.6 (OS)**	1.3	1.0	n.d.	n.d.	n.d.	0.3	n.d.	n.d.	n.d.	Hypofunction L HC (utricle and saccule not tested)
#6 (4 months)	L	**0.6** **(CS, OS)**	1.1	**0.6** **(CS,OS)**	−0.14	–**1**	–**1**	0.4	0.9°	**HSN R** (6°/s), **VIN R** (6°/s)	–**80%**	Hypofunction L HC, PC and saccule
#7 (1 month)	R	1.0 **(CS, OS)**	0.8 **(CS)**	0.9	0.0	0.28	n.d.	0.5	**7.5**°	**SN L** (3°/s), **HSN L** (6°/s)	**79%**	Hypofunction R HC, (AC) and utricle
#7 (3 months)	R	1.2	1.2	1.1	−0.16	0.05	n.d.	0.4	1.2°	No nystagmus	**45%**	Recovery of R vestibular function apart from CP
#8 (1 month)	L	1.0 **(CS)**	0.8	0.8	−0.26	−0.37	0.03	0.4	–**2.5**°	No significant nystagmus	–**52%**	Hypofunction L HC and utricle

*vHIT gains and DVA values are presented for the affected side only (within normal range on the unaffected side for all patients). Pathological values are printed in bold (see “PATIENTS AND METHODS” section for definition of normal values and further details). For o- and cVEMP ARs, SVV and CP, positive values show relative hypofunction on the right side, while negative values indicate relative left-sided hypofunction*.

*vHIT, video head impulse test; HC, horizontal canal, AC, anterior canal; PC, posterior canal; o-/c-VEMP, ocular / cervical vestibular evoked myogenic potentials; AR, asymmetry ratio; ACS, air-conducted sound; BCV, bone-conducted vibration; DVA, dynamic visual acuity; SVV, subjective visual vertical; VOG, video-oculography; SPV, slow-phase velocity; CP, caloric paresis; R, right; L, left; n.d., not determined; VIN, vibration-induced nystagmus; CS, covert saccades in vHIT; OS, overt saccades; HSN, head shake nystagmus; SN, spontaneous nystagmus*.

#### Semicircular Canal Function

High-frequency function for all three semicircular canals (SCCs) was assessed by the video head impulse test (vHIT; Otometrics, Natus Medical Denmark, Taarstrup, Denmark) as described previously (34). SCC hypofunction was defined by a reduced vestibulo-ocular reflex (VOR) gain (<0.8 for the horizontal canal (HC) and <0.7 for the anterior (AC) and posterior (PC) canals) with the additional presence of corrective saccades (35–37). Repetitive vHIT measurements during the recovery process of AUPVP have revealed that corrective saccades may still be present after VOR gain values have already normalized (38, 39). Therefore, we also analyzed the presence of covert saccades (CS) and overt saccades (OS) for all SCCs tested (Table 3).

In addition, the response of the horizontal SCCs to low-frequency vestibular stimuli was evaluated by caloric irrigation of the external ear canals with warm (44°C) and cold (30°C) water (Atmos Variotherm plus, Lenzkirch, Germany) (40–42). A caloric paresis (CP) factor >25% calculated by the Jongkees formula (43) was employed as a measure for relative unilateral caloric hypofunction. In the present study, positive values represent right-sided and negative values left-sided caloric hypofunction (Table 3).

Dynamic visual acuity (DVA) was used to determine functional integrity of the VOR of the horizontal SCCs (44, 45). In detail, visual acuity was measured in logMAR (decadic logarithm of the mean angular resolution), with the DVA value representing the decrement from static to dynamic visual acuity. Abnormal DVA values were determined by comparison with age-related normative values from our lab.

Nystagmus analysis was performed with video-oculography (VOG) in the dark (Interacoustics, Middelfart, Denmark) (40, 42). Nystagmus direction was defined by the direction of the quick phase. The presence of a spontaneous nystagmus (SN) with a slow phase velocity (SPV) >3°/s or a head-shaking nystagmus (HSN) in the same direction as the SN were interpreted as signs of incomplete central compensation of SCC function after AUPVP (42). Vibration-induced nystagmus (VIN) was tested by applying a 100 Hz vibration stimulus (VestiVIB, Autronic, Hamburg, Germany) to either mastoid as described by Dumas et al. (46). Presence of a VIN >2.5°/s SPV indicated asymmetrical SCC function between the right and the left side with the quick phase of the nystagmus beating toward the side with the higher vestibular activity (47). Positional nystagmus was evaluated during the Dix-Hallpike maneuver and the supine roll maneuver to either side using either VOG or Frenzel goggles.

#### Otolith Function

Dynamic (transient) utricular and saccular function was tested by ocular and cervical vestibular evoked myogenic potentials (o- and cVEMPs), respectively, using an Eclipse platform (Interacoustics, Middelfart, Denmark) with the stimulation parameters and recording procedures described by Tarnutzer et al. (48). An asymmetry ratio (AR) ≥0.3 indicated asymmetrical otolith function for both o- and cVEMPs based on normative values of our lab. In Table 3, a positive AR indicates right-sided relative hypofunction, and a negative AR left-sided relative hypofunction.

Cervical VEMPs were primarily recorded in response to monaural air-conducted sound (ACS) at 500 Hz and 100 dB normal hearing level (nHL) presented *via* headphones (Telephonics TDH-39P; Telephonics Corp., Farmingdale, NY, USA). ACS cVEMPs are very sensitive to middle ear dysfunction with air-bone gaps as small as 8.75 dB able to diminish or cancel the response (49). Therefore, we performed an additional cVEMP recording using bone-conducted vibration (BCV) to Fz (midline of the forehead at the hairline) in those patients with asymmetrical ACS cVEMP responses in order to determine whether a reduced cVEMP amplitude was rather due to middle ear dysfunction (asymmetrical ACS cVEMPs and symmetrical BCV cVEMPs) or saccular hypofunction (asymmetrical ACS and BCV cVEMPs), as described before (48). Dynamic saccular hypofunction was diagnosed if both ACS and BCV cVEMPs yielded an AR ≥0.3. The BCV stimulus was delivered to Fz by a powerful Minishaker (4810, Bruel and Kjaer, Naerum, Denmark) connected to an amplifier (2718, Bruel and Kjaer). Cervical VEMP p13n23 amplitudes were normalized to the muscular background activity of the ipsilateral sternocleidomastoid muscle, resulting in unitless values for corrected cVEMP amplitudes [see (50) for details]. Measurements were discarded if the background muscular activity was <60 μV.

For oVEMPs, only 500 Hz BCV stimuli were applied to Fz by the Minishaker. Due to their excitatory nature, oVEMP n10p15 amplitudes do not have to be normalized to background muscular activity of the contralateral inferior oblique muscle and are therefore measured in μV.

Static (sustained) utricular function was determined by measuring the subjective visual vertical (SVV), as described recently (51). Based on normative values of our lab, values up to ±2.2° were judged as normal (positive value: deviation to the right, negative: deviation to the left). SVV and oVEMP amplitudes/AR do not necessarily correlate as they assess two different channels of otolith function: while oVEMPs probe dynamic (transient) utricular function mainly mediated by type I vestibular hair cells and irregular utricular afferents of the striola, the SVV is an indicator of static (sustained) utricular function determined mainly by peripheral type II vestibular hair cells and regular utricular afferents (51, 52).

### Magnetic Resonance Imaging

Two patients (#1 and #7) underwent magnetic resonance imaging (MRI) of the brain and the temporal bone mainly for exclusion of acute stroke 5 days (#1) and 1 day (#7) after the onset of the acute vestibular syndrome. Acquisition and interpretation of the images were performed by the Department of Neuroradiology at Zurich University Hospital. All MR images were acquired on a three Tesla MRI Scanner (Skyra, release E 11, Siemens Healthcare, Erlangen, Germany). The MRI protocol consisted of diffusion-weighted imaging (DWI), fluid-attenuation inversed recovery (FLAIR), T2-weighted (T2w) und T1 non-contrast- and contrast-enhanced sequences. Intravenous Dotarem (gadoterate meglumine, 0.5 mmol/ml) at a concentration of 0.2 ml/kg body weight was used as a contrast agent.

In detail, DWI [acquisition type 2D, read-out segmented echo planar imaging approach, field of view 220 × 220 (mm^2^), number of slices 38, voxel size 1.1 × 1.1 × 3.0 (mm^3^), slice gap 0.9 (mm), repetition time (TR) 7,340 (ms), echo time (TE) 68 (ms), *B*-values 0 respectively 1,000 (s/mm^2^), acquisition time (TA) 4:33 (min:s)], FLAIR [acquisition type 3D, field of view 240 × 233 (mm^2^), number of slices 176, voxel size 0.5 × 0.5 × 1.0 (mm^3^), TR 4,700 (ms), TE 386 (ms), inversion time (TI) 1,530 (ms), TA 5:59 (min:s)], and T2w [acquisition type 2D, turbo-spin echo approach (TSE), field of view 220 × 220 (mm^2^), number of slices 44, voxel size 0.4 × 0.4 × 3.0 (mm^3^), slice gap 0.3 (mm), TR 8,180 (ms), TE 100 (ms), TA 3:26 (min:s)] sequences were acquired in transverse orientation.

Furthermore, time-of-flight (TOF) and contrast-enhanced (CE) magnetic resonance angiography (MRA) of cerebral vessels were accomplished with the following parameters: TOF [acquisition type 3D, field of view 190 × 175 (mm^2^), number of slices 240, voxel size 0.3 × 0.3 × 0.6 (mm^3^), slice gap −4.8 (mm), repetition time (TR) 21.0 (ms), echo time (TE) 3.43 (ms), Flip Angle 20 Deg., TA 5:37 (min:s)]; CE-MRA [acquisition type 3D, field of view 325 × 310 (mm^2^), number of slices 88, voxel size 0.8 × 0.8 × 0.9 (mm^3^), slice gap −0.18 (mm), repetition time (TR) 3.29 (ms), echo time (TE) 1.26 (ms), Flip Angle 25 DEg, TA 0:22 (min:s)].

## Results

### Patient Demographics

Within the study period, *n* = 8 patients met the inclusion criteria (diagnosis of AUPVP and symptom onset within the first 30 days after COVID-19 vaccination). The mean interval between the last dose of the vaccine and the beginning of symptoms was 11.9 days (SD 4.8) with a median of 10 days and a range between 6 and 20 days. The demographic data of the patients are summarized in Table 1: two were female, six were male, the mean age at onset of symptoms was 46 years (SD 11.7). All patients had received mRNA vaccines (*n* = 7: Moderna, *n* = 1: Pfizer/BioNTech). Three patients developed AVS after the first dose, three after the second, and two after the third (“booster”). Of note, all patients experienced only one episode of acute vestibular symptoms, even those who had received more than one vaccination dose (*n* = 5).

### Vestibular Testing

On initial bedside examination, all patients showed a horizontal or horizontal-torsional SN with the quick phase beating toward the contralateral ear following Alexander's law and a positive h-HIT toward the affected side (Table 2). For patients #2 and #6, this information was obtained from the medical records of the referring physicians. None of the patients displayed signs and symptoms of any otological disease, central vestibular syndrome or acute herpes zoster.

**Figure 1:**
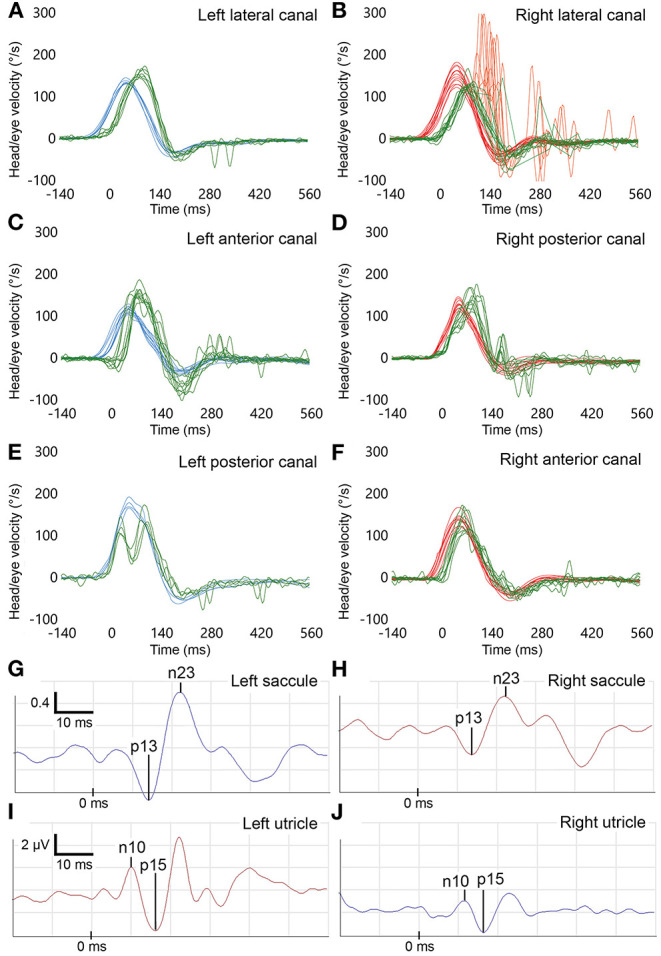
Vestibular function tests in patient #2 (right superior vestibular nerve or its endorgans affected). **(A–F)** Video head impulse test (vHIT) results of all six semicircular canals. Eye velocity is right-left mirrored for better comparison with head velocity. **(A,C,E)** Head impulses stimulating left-sided semicircular canals. Blue traces: head velocity. Green traces: eye velocity of the vestibulo-ocular reflex. **(B,D,F)** Head impulses stimulating right-sided semicircular canals. Red traces: head velocity. Green traces: eye velocity of the vestibulo-ocular reflex. Red traces superimposed on green traces: catch-up saccades. The eye velocity traces indicate hypofunction of the right lateral semicircular canal (gain = 0.7). **(G,H)** Cervical vestibular-evoked myogenic potentials (cVEMPs) in response to air-conducted sound. *Y*-axis indicates the normalized p13n23 amplitude (unitless). The traces show slightly reduced cVEMP responses for the right (red traces) as compared to the left saccule (blue traces), which are still within normal range (asymmetry ratio, AR = 0.26). **(I,J)** Ocular vestibular evoked myogenic potentials (oVEMPs). *Y*-axis indicates absolute amplitude (μV). The response of the right utricle (blue traces – crossed reflex pathway) is smaller compared to the left side (red traces), AR = 0.42. *X*-axis represents time in all graphs.

**Figure 2:**
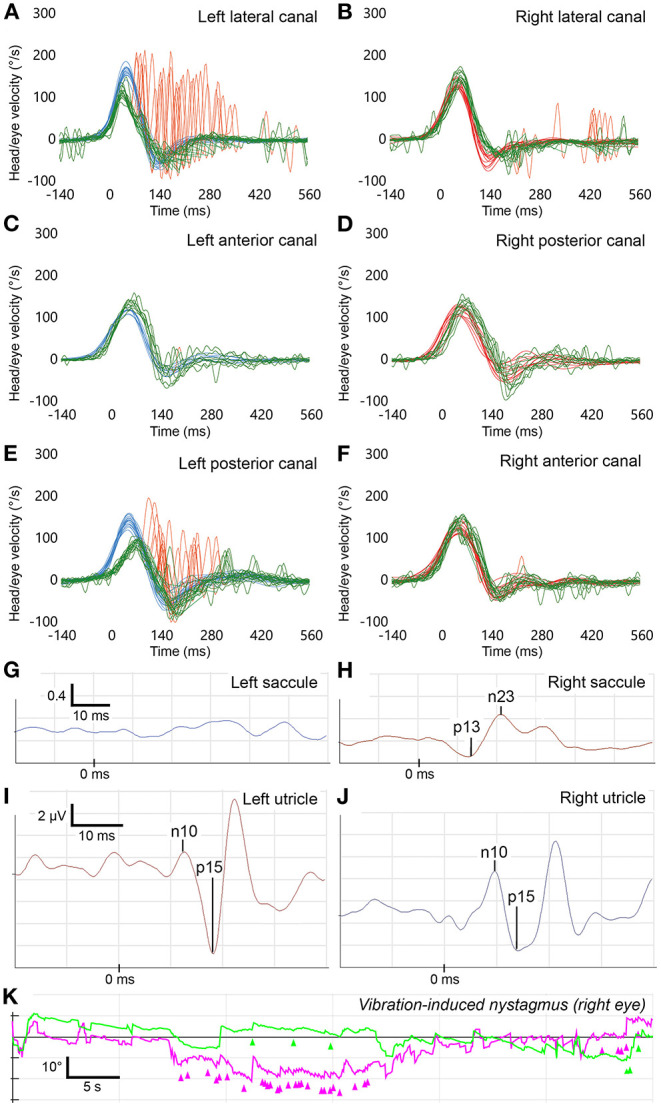
Vestibular function tests in patient #6 (left superior and inferior vestibular nerves or their endorgans affected). See legend of Figure 1 for general features of **(A–J)**. The vHIT traces indicate hypofunction of the left lateral and posterior semicircular canals (gain = 0.6 each), loss of the cVEMP response of the left saccule (AR = −1) and symmetrical oVEMP responses (AR = −0.14). **(K)** Video-oculography recordings during a 100 Hz vibration stimulus applied to the left mastoid. The magenta trace shows horizontal eye position (°) and the green trace shows vertical shows eye position (°) as indicated on the *y*-axis. Nystagmus quick phases are labeled with arrow heads. The right-beating vibration-induced nystagmus is consistent with hypofunction of the left lateral semicircular canal. *X*-axis represents time in all graphs.

Table 3 summarizes the results of laboratory vestibular testing at a 1- to 5-month interval after symptom onset. Representative examples are shown in Figures 1, 2. Patient #7 was examined 1 and 3 months after the acute event, while all the other patients were tested only once. Two patients (#1 and #3) displayed no more signs of unilateral vestibular hypofunction after 1 month and reported complete resolution of symptoms. Taking the initial clinical findings from Table 2 into account (positive h-HIT to the affected side and horizontal-torsional SN to the unaffected side, we concluded that their vestibular function had recovered. Patient #1 yielded a very mild recovery VIN (fast phase directed to the originally affected right side) with a slow-phase velocity of 3°/sec (see Discussion).

For the remaining six patients, the following vestibular endorgans were affected by AUPVP in declining order: HC (*n* = 6), utricle (*n* = 4), PC (*n* = 1), saccule (*n* = 1). Dynamic saccular hypofunction confirmed by both asymmetrical ACS and BCV cVEMPs was detected in only one patient (#6). Left-sided dynamic saccular function was interpreted as normal in patient #8 due to his symmetrical BCV cVEMP response (AR = 0.03). Two patients (#4 and #5) did not undergo o- and cVEMP testing, so we have no information of dynamic utricular and saccular function in them. Only one patient (#7) displayed a possible transient hypofunction of the AC: while corrective covert saccades (CS) with a normal gain (0.8) were present at 1 month after symptom onset, these were no longer detectable after 3 months, indicating recovery of the anterior canal. The endorgan involvement (AC, HC, utricle) of this patient at 1 month was compatible with a selective damage to the supply area of the superior vestibular nerve, while the other five subjects with a vestibular deficit in laboratory testing yielded a heterogeneous pattern of endorgan involvement not fitting exactly with the supply areas of the superior and/or inferior vestibular nerves (Table 3). This aspect will be dealt with in detail in the discussion.

### MRI Examination

Two patients underwent MRI examination of the brain and the temporal bone in the acute stage of the disease (patient #1 5 days and patient #7 1 day after the onset of symptoms). In patient #1, no T1 hyperintensity was detected in the right inner ear and the internal auditory canal before and after administration of gadolinium, thus showing no signs of hemorrhage or inflammatory disease. For patient #7, only non-contrast-enhanced sequences were available. Again, there were no signs of right-sided inner ear hemorrhage in T1. In addition, no hyperintensities indicating inflammation were detected in the right inner ear and the internal auditory canal in the FLAIR sequence.

### Treatment

All patients received initial symptomatic treatment (i.e., antiemetics and intravenous fluid) and vestibular rehabilitation therapy. All but two patients (#5 and #6) were treated with oral and/or intravenous steroids in the acute phase of the disease. In detail, patients #1 and #7 received one shot of methylprednisolone 1g i.v., followed by prednisone p.o. 100 mg for 3 days and consecutive reduction of the dose by half every 3 days Patients #2, #3 and #4 obtained 50 mg of prednisone p.o. for 5 days.

## Discussion

To the best of our knowledge, this is the first study analyzing the occurrence of AUPVP after COVID-19 vaccination in a specialized neurotology center, identifying eight patients within 7 months. The findings of the study are discussed with special regard to the estimated incidence of AUPVP after COVID-19 vaccination, possible underlying pathomechanisms, treatment, strengths/limitations of the study and perspectives for future investigations.

### Estimated Frequency of Acute Unilateral Peripheral Vestibulopathy

In total, 26 patients with new-onset AUPVP presented to our institution within the 7 months of the study period, which would correspond to a 1-year-incidence of 3/100,000 for the Kanton Zurich (1.56 million inhabitants), our main catchment area. Of these 26 patients, 31% (*n* = 8) developed acute vestibular symptoms within 30 days after COVID-19 vaccination.

These numbers reflect a certain selection bias for our institution: as we are a tertiary neurotology center, many patients with acute vestibular symptoms are seen by other healthcare providers of the catchment area, which explains the low overall estimated incidence of AUPVP calculated from the numbers of our center as compared to the literature (1-year-incidence of 3.5 to 24/100,000) (53). On the other hand, particularly patients with severe symptoms or complex medical issues are referred to our institution, which may account for the high percentage of patients with AUPVP after COVID-19 vaccination (31%). Considering the 542,860 people who received at least one vaccination dose in the Kanton Zurich within the study period (54), our data suggest 1.47 cases of AUPVP per 100,000 persons within 30 days after vaccination, indicating a low risk. Further possible confounders of the study are discussed in Section Implications For Treatment and Further COVID-19 Vaccinations.

### Types of Vaccine

All patients of the present study had received an mRNA vaccine. This does *not* reflect any potential relationship between the type of the vaccine and the occurrence of AUPVP, but is rather a result of local availability. As of 30 March, 2022, only three different vaccines have been authorized in Switzerland: the mRNA vaccines by Pfizer/BioNTech (authorized on 19 Dec 2020) and Moderna (authorized on 12 Jan 2021) and the vector-based vaccine by Janssen (authorized on 22 Mar 2021) (55). In addition, the Swiss health authorities generally recommend mRNA vaccines (56), so that the latter account for >99% of the 8,862,579 million vaccination doses administered in Switzerland within the study period (67.6% Moderna, 31.8% Pfizer), while the vector-based vaccine comprises only 0.6% of vaccination doses (57). Thus, the predilection of AUPVP in patients immunized with the Moderna vaccine rather reflects the distribution of vaccines in the general population than a possible causal relationship.

### Interpretation of Results

#### Results From Selected Patients

Patient #1 displayed a subtle VIN beating toward the initially affected right side (SPV = 3°/s, normal values up to 2.5°/s) on VOG recording 1 month after the onset of symptoms (Table 3). At this time, the initial SN to the left and the pathological h-HIT to the right (Table 2) had recovered. Therefore, we interpret the subtle VIN to the right as a recovery nystagmus caused by the persistence of a certain degree of central compensation for an initial imbalance in vestibular tone after the need for this amount of compensation was no longer present due to recovery of peripheral vestibular function (58, 59). Similar observations have been made before for patients in the compensation/recovery phase after AUPVP: Dumas et al. (60) described a VIN with the quick phase beating toward the affected side in 11% of patients in the late phase of AUPVP. Likewise, Park et al. (61) reported that an initial paralytic (i.e., beating toward the unaffected side) VIN reversed its direction after 2 months in 10% of patients with AUPVP.

Patient #7 underwent vestibular laboratory testing 1 and 3 months after the onset of symptoms (Table 3). After 1 month, the right-sided horizontal and anterior canals displayed refixation saccades, but normal VOR gain values (1.0 for the HC and 0.8 for the AC). On the second assessment 2 months later, these saccades had disappeared. At the same time, the SVV had recovered, no spontaneous and head-shaking nystagmus was detected in the VOG, and caloric paresis of the horizontal canal had recovered from initially 79 to 45%. In summary, these results indicate an ongoing recovery of right-sided vestibular function. The transient presence of refixation saccades despite a normal VOR gain during the compensation/recovery process of AUPVP is in line with the findings reported by MacDougall and Curthoys (62), Manzari et al. (38) and Yang et al. (39). The consecutive vHIT measurements in patients recovering from AUPVP from these studies showed that VOR gain may recover earlier than refixation saccades. As the first vHIT examination was performed 1 month after the onset of symptoms in patient #7, we might well have missed an initial reduction of VOR gain.

A second noteworthy finding in patient #7 is the different time course for recovery of high-frequency (vHIT) and low-frequency function (caloric testing) of the horizontal canal: while vHIT testing displayed only refixation saccades with a normal VOR gain 1 month after symptom onset, there was a high degree of caloric asymmetry (CP = 79%) at that time, which improved up to a CP of 45% within the next 2 months (Table 3). This observation is in line with the findings by Zellhuber et al. (63) who attributed the different time courses of HC function recovery as measured by vHIT and caloric testing to the two different aspects of horizontal canal function (high- and low-frequency) assessed by these two tests.

#### Involvement of Individual Vestibular Endorgans

In summary, hypofunction was detected more frequently for endorgans supplied by the superior vestibular nerve (HC: *n* = 6, utricle: *n* = 4) than those of the inferior vestibular nerve (PC: *n* =1, saccule: *n* = 1), which is in line with the distribution found by Taylor et al. (15). Comparison between our study and the latter is however limited by two facts: first, vestibular laboratory testing was performed at different timepoints [Taylor et al. (15): within 10 days after symptom onset; present study: 1–5 months after symptom onset]. Therefore, we might have missed initial hypofunction of vestibular endorgans: for instance, the anterior canal showed a reduced VOR gain for 90.7% of patients in Taylor's study (15), while we only detected corrective saccades with a normal VOR gain in one (#7) out of eight patients in the present study. Second, two patients in the present study did not undergo o- and cVEMP testing, so that we cannot make a definite statement about the number of patients affected by dynamic utricular and saccular hypofunction.

There has been a debate in the literature whether all endorgans supplied by one branch of the vestibular nerve must be affected to the same extent in order to qualify for the diagnosis of vestibular neuritis, i.e., a damage to the vestibular nerve rather than the vestibular labyrinth. Uffer and Hegemann (16) reported that this was the case in only 24% of patients with AUPVP tested within 10 days of symptom onset. In the present study, only one (patient #7) out of eight patients (12.5%) displayed an involvement of all endorgans supplied by the superior vestibular nerve (HC, AC and utricle) after 1 month. Again, comparability between our results and the ones by Uffer and Hegemann (16) is limited by the different timepoints of vestibular testing. We found no case where only endorgans of the inferior vestibular nerve were affected, which is in line with the low proportion of an isolated AUPVP of the inferior vestibular nerve and / or its endorgans (saccule, posterior canal) reported in the literature (1.2%−5%) (64).

To date, it is still elusive whether AUPVP is primarily a disorder of the vestibular labyrinth, the vestibular nerve or both (17). In the present study, brain MRIs from the acute phase of the disease were available for two patients (#1 and #7) indicating neither a structural lesion in the vestibular labyrinth or the vestibular nerve. It should, however, be noted that these MRIs were primarily done for detection of stroke and not for evaluation of the inner ear/the internal auditory canal.

### Possible Underlying Mechanisms of AUPVP Following COVID-19 Vaccination

Onset of acute vestibular symptoms within 6 to 20 days after administration of a COVID-19 vaccine in the present study is in line with the temporal course of HSV reactivation and autoimmune neurological disorders occurring after vaccination against SARS-CoV-2.

#### Herpes Simplex Virus Reactivation

A growing body of evidence from anatomical, immunohistological, molecular biological and genetic studies suggests a reactivation of herpes simplex virus hibernating in the neurons of the vestibular ganglia as a possible cause for AUPVP (65–69). Reactivation of latent HSV and VZV infections including dermatological and ophthalmological manifestations has been observed within 1–16 days after COVID-19 vaccinations with different types of vaccine (8, 28–31). A review of 40 dermatological cases described a median latency of 13 days between vaccination and onset of clinical symptoms for HSV reactivation (31), which is in line with the time course observed in the present study (mean latency: 11.9 days, median: 10 days). Reactivation of HSV or VZV is not unique for COVID-19 vaccinations, but has been observed before for hepatitis A and influenza vaccines (70). While a causal relationship has not been proven, immune-modulatory effects of the vaccine are discussed in this context, in particular suppression of cellular immunity (28) by inhibition of distinct Th1 cell populations (8, 29). Reactivation of herpes virus is also supposed to play a role in facial nerve palsy after COVID-19 vaccination (8).

#### Autoimmune Response

Both mRNA and vector-based vaccines induce an immune response of the host against the spike glycoprotein of SARS-CoV-2 (3). Of note, there is a high degree of structural homology between this protein and the human proteome (molecular mimicry), which may cause a cross-reaction of the vaccine-induced immune response against self-proteins resulting in autoimmune disorders after COVID-19 vaccination (71, 72). Molecular mimicry as an underlying cause for autoimmune reactions in temporal association with vaccinations has been discussed before (e.g., for hepatitis B, influenza or human papillomavirus vaccination) (73), and new-onset autoimmune disorders have been observed after COVID-19 vaccinations, e.g., immune thrombotic thrombocytopenia and Guillan-Barré syndrome (7). A recent study found a median of 11 days between vaccination and onset of autoimmune neurological disorders (32), which corresponds to the start of IgG production between 10 and 14 days after vaccination (9, 74).

The inner ear might also be a target of autoimmune cross-reactivity following COVID-19 vaccination, as its proteome shares immunogenic heptapeptides with the SARS-CoV-2 protein (e.g., peptide sequences of prestin and wolframin) (72). This notion is supported by a recent description of autoimmune inner ear disease (AIED) with bilateral sensorineural hearing loss and bilateral vestibulopathy following COVID-19 vaccination with the Pfizer mRNA vaccine (27).

#### Summary

The results from the present study do not provide evidence for a specific underlying pathophysiology of AUPVP after COVID-19 vaccination. No patient displayed clinical signs for HSV reactivation (e.g., skin lesions, HSV keratitis) or a systemic autoimmune disorder. Furthermore, the MRIs of the brain and temporal bone in patients #1 and #7 did not indicate a structural lesion of either the vestibular labyrinth or the nerve. Regarding the endorgan lesion patterns in laboratory vestibular testing, a disorder of the superior vestibular nerve - e.g., by reactivation of HSV in the vestibular ganglia - may be suspected in patient #7. All the other patients showed a “patchy” involvement of individual vestibular endorgans that did not fit with a selective damage to the superior or inferior vestibular nerve, which might be due to an autoimmune response against antigens of the vestibular labyrinth or autoimmune vasculitis (17).

### Implications for Treatment and Further COVID-19 Vaccinations

Six of eight patients in the present study received systemic steroids in the acute phase of the disease. In retrosptect, this treatment must be re-evaluated critically regarding a potential attenuation of the vaccine-induced immune response against SARS-CoV-2 (12, 75, 76). When the patients presented with acute vestibular symptoms, they usually did not mention that they had received a recent COVID-19 vaccination, and the question about vaccination status was not part of our clinical routine before this study. In the meanwhile, this question has been added to our standardized questionnaire.

To date, it is not clear how systemic corticosteroids affect the immunogenicity of different COVID-19 vaccines. In accordance with guidelines for other inactivated vaccines, it is recommended to wait for at least 2 weeks after vaccination before a high-dose course of systemic steroids is started [Soy et al. (76) and personal information: Dr. Nadia Eberhard-Kuhn, Department of Infectious Diseases and Hospital Epidemiology, University Hospital Zurich]. As there is currently no high-quality evidence for the efficacy of systemic corticosteroids in AUPVP (77, 78), the potential risks and benefits of starting a treatment should be balanced for each single patient. Consulting an immunologist or infectious disease specialist is useful in this context. As described for SSNHL after COVID-19 vaccinations before (9, 12, 75), intratympanic application of steroids into the affected ear is also an alternative (17).

Our patients were often hesitant to obtain the next vaccination dose. No official recommendations are currently available for such situations. As the risk of contracting a SARS-CoV-2 infection with all its possible complications outweighs the risk of experiencing another episode of acute vestibular syndrome, we encourage our patients to proceed with vaccination. We recommend changing the type of vaccine if possible and to take valaciclovir for 7 days starting 2 days before the vaccination to suppress HSV reactivation (9, 27). Patients are furthermore advised to contact us immediately if they experience vertigo symptoms, so we can diagnose a possible relapse of vestibular dysfunction and start treatment (e.g., by intratympanic steroid injection) as soon as possible.

### Strengths, Limitations, and Perspectives

This is the first case series of patients developing AUPVP within 1 month after COVID-19 vaccination. The strength of the study is that all patients underwent a comprehensive bedside neurotological examination at onset of symptoms and that laboratory vestibular testing was performed in all patients.

The major limitation of the study is its retrospective nature. As patients were not included prospectively into the study, not all bedside and laboratory tests were available for all patients. Furthermore, vestibular laboratory examination was only conducted 1 month after the onset of symptoms in most patients because this is part of our routine clinical protocol for AUPVP. This delay had no impact on the diagnosis of AUPVP in the present study because the disorder was defined by clinical criteria. In future prospective studies, laboratory vestibular testing of all five vestibular endorgans should, however, be performed within the first 10 days of symptom onset in order to grasp the full extent of initial vestibular hypofunction and to achieve a better comparability with previous studies on this topic (15, 16).

Another limitation of the study is a possible selection bias of patients who were recruited from a single tertiary neurotology center. On the one hand, the number of patients with AUPVP after COVID-19 vaccination may have been underestimated because not all patients were routinely asked about their vaccination status at the time of the study; on the other hand, a possible temporal association between the two events may have been overestimated due to the high number of 542,860 people in the Kanton Zurich (approximately one third of the inhabitants) who received at least one COVID-19 vaccination during the study period, which makes it more likely that any medical condition, including AUPVP, occurs in temporal association with a vaccination.

To obtain more representative and reliable results about a possible link between COVID-19 vaccination and the rare event of an AUPVP, large-scale, multi-center studies are warranted. In this context, self-controlled case series (SCCS) offer a valuable approach because each subject acts as its own control, thus minimizing the effect of possible confounders (6). Furthermore, these studies should comprise follow-up vestibular laboratory testing in order to determine the prognosis of AUPVP after COVID-19 vaccination as compared to other AUPVP cases. In the present study, results from repetitive vestibular testing were only available for patient #7 (1 and 3 months after the acute event, Table 3) indicating a recovery of right-sided vestibular function apart from caloric paresis of the right horizontal canal.

## Conclusion

This is the first study to report a temporal association between COVID-19 vaccination and AUPVP in several cases of one tertiary neurotology center. Based on our results, the risk of AUPVP within 30 days after the vaccination is very low. Nevertheless, we recommend to ask patients with a new diagnosis of AUPVP about the date of their last vaccination and the type of vaccine they received. Although a causal relationship is not known to date, cases should be reported to health authorities in order to provide data for future epidemiological investigations on possible side effects of COVID-19 vaccination.

## Data Availability Statement

The raw data supporting the conclusions of this article will be made available by the authors, without undue reservation.

## Ethics Statement

A formal ethical approval was waived by the local Ethics Committee (Kantonale Ethikkommission, Zurich, Switzerland) in view of the retrospective nature of the study, which is based on single cases, and since all the procedures being performed were part of the routine care. The patients/participants provided their written informed consent to participate in this study.

## Author Contributions

JD and DS designed the study. MS, DB, DS, and JD collected and analyzed the data. DB created the figures. AP interpreted the MR images. MS and JD wrote a first draft of the manuscript. All authors contributed to manuscript revision, read, and approved the submitted version.

## Funding

DB was supported by a national MD-PhD scholarship from the Swiss National Science Foundation.

## Conflict of Interest

The authors declare that the research was conducted in the absence of any commercial or financial relationships that could be construed as a potential conflict of interest. The reviewer SH declared a shared parent affiliation with the authors to the handling editor at the time of review.

## Publisher's Note

All claims expressed in this article are solely those of the authors and do not necessarily represent those of their affiliated organizations, or those of the publisher, the editors and the reviewers. Any product that may be evaluated in this article, or claim that may be made by its manufacturer, is not guaranteed or endorsed by the publisher.
